# Early motor trajectories and distinct psychopathological profiles in adolescents born very and extremely preterm

**DOI:** 10.3389/frcha.2026.1894705

**Published:** 2026-08-31

**Authors:** Emanuela Claudia Turco, Davide Fausto Borrelli, Federica Cimmino, Laura Caiazza, Lorenzo Petrolini, Tommaso Vitali, Cecilia Ferrari, Benedetta Piccolo, Susanna Esposito, Maria Carmela Pera, Matteo Tonna

**Affiliations:** 1 Child Neuropsychiatric Unit, Maternal and Child Health Department, Parma University-Hospital, Parma, Italy; 2 Department of Medicine and Surgery, Psychiatric Unit, University of Parma, Parma, Italy; 3 Department of Mental Health, Local Health Service, Piacenza, Italy; 4 Department of Biomedical, Metabolic and Neural Sciences, University of Modena and Reggio Emilia, Modena, Italy; 5 Department of Medicine and Surgery, Pediatric Clinic, University of Parma, Parma, Italy; 6 Department of Medicine and Surgery, Child Neuropsychiatry Unit, University of Parma, Parma, Italy; 7 Department of Mental Health, Local Health Service, Parma, Italy

**Keywords:** autism spectrum disorder, neurodevelopmental disorders, preterm birth, psychosis, social functioning

## Abstract

**Background:**

In recent decades, advances in neonatal care have led to a substantial reduction in mortality associated with prematurity. However, preterm birth remains the leading contributor to long-term neurodevelopmental impairment across the lifespan. The present study aimed to longitudinally assess neurodevelopmental outcomes in individuals born very preterm (VPT) or extremely preterm (EPT) and to retrospectively investigate associations between early neurodevelopmental trajectories and psychopathological and functional profiles in adolescence.

**Methods:**

The study included 52 adolescents aged 14–18 years, drawn from an original cohort of 118 individuals born before 31 weeks of gestation between 2005 and 2011. Neonatal clinical and anamnestic data were collected, and early neurodevelopment within the first two years of life was assessed using the Bayley Scales of Infant and Toddler Development, Second Edition. Fifteen years later, participants underwent a multidimensional follow-up assessment via self-administered questionnaires delivered through a secure web-based platform. Both participants and one parent completed a comprehensive psychopathological evaluation, including the Child Behavior Checklist (CBCL), Autism Spectrum Quotient (AQ), Obsessive–Compulsive Inventory (OCI), Prodromal Questionnaire–16 (PQ-16), and Inventory of Psychotic-Like Anomalous Self-Experiences (IPASE).

**Results:**

The mean gestational age was 28 weeks (SD = 1.8; range: 23–31), and the mean birth weight was 1,120.8 g (SD = 322.4). VPT individuals showed higher AQ scores, suggesting increased vulnerability to autistic traits, whereas EPT individuals exhibited higher CBCL Attention Problems scores, consistent with ADHD-related features. Latent Profile Analysis identified a two-profile solution: Profile 1 (“High autism traits”) was characterized by elevated AQ scores and relatively low OCI, PQ-16, and IPASE scores, whereas Profile 2 (“High psychotic and OCD traits”) showed markedly elevated PQ-16, IPASE and OCI scores. Hierarchical multiple regression analyses revealed that the Psychomotor Development Index slope was the only significant predictor of later social functioning (B = −5.782, *β* = −.289, *p* = .040), with more favorable early motor trajectories associated with fewer social difficulties in adolescence.

**Conclusions:**

Very and extremely preterm birth are associated with long-term neurodevelopmental and psychopathological vulnerability. Early motor development appears to be a key predictor of later social functioning, potentially shaping distinct developmental trajectories toward autism-related features or psychosis vulnerability.

## 1 Introduction

In 2020, about 13.4 million babies were born preterm (9.9% of all births), with 15% occurring before 32 weeks of gestation—4.2% before 28 weeks (EPT) and 10.4% between 28 and 32 weeks (VPT) (1). Preterm birth accounts for around 1 million neonatal deaths and was the leading cause of mortality in 2022 (2).

Despite advances in neonatal care reducing mortality, prematurity remains the main cause of long-term neurodevelopmental impairment. It is in fact associated with increased risks of motor, cognitive, sensory, and behavioral disorders, with effects that can persist into adulthood (3). For instance, a Norwegian cohort study following over 900,000 individuals found that those born extremely preterm had significantly higher risks of cerebral palsy, intellectual disability, and reliance on disability pensions in adulthood (4). Similarly, a German study of adolescents born before 27 weeks reported higher rates of psychological problems and lower health-related quality of life (5).

Preterm birth has consistently been associated with an increased risk of psychiatric disorders (6–10), and psychiatric hospitalization has been shown to be significantly associated with the degree of prematurity (9, 11). Most studies have concentrated on developmental disorders emerging during childhood, such as Attention-Deficit/Hyperactivity Disorder (ADHD) and Autism Spectrum Disorder (ASD). In this regard, Johnson and Marlow (12) support the proposal of a “preterm behavioral phenotype” for describing a set of symptoms including inattention, anxiety and social difficulties, cutting across different diagnoses (e.g., ADHD, ASD and Anxiety Disorder), that in very preterm population is up to ten times higher compared to term population. With regard to ASD, Crump et al. (13) support a potential causal relationship between preterm birth and ASD that is only partially explained by shared familial factors, suggesting that reduced gestational age itself contributes to the risk. However, only few studies have explored the vulnerability to adult psychopathology (6, 12), despite the strong evidence of a 3- to 4-fold risk of developing psychiatric disorders in preterm-born children compared to controls (12, 14). In this regard, some authors (15) analyzed self-reports of adults born very and extremely preterm, showing a heightened risk for internalizing problems and socially avoidant personality traits along with a lowered risk for externalizing problem types. Among these, social functioning—defined as the ability to understand and adapt behaviour and emotions to social contexts and to facilitate interpersonal relationships (16) represents an important long-term outcome following very preterm birth (17). Adolescents born VPT or EPT show greater difficulties in social functioning compared with their full-term peers (7, 17, 18), even when individuals with intellectual disabilities and neurosensory disorders are excluded. Self-reports from EPT adolescents indicate fewer friendships, less time spent with peers, greater dissatisfaction with their social networks, and higher rates of bullying compared with FT peers (19). These difficulties often persist into adulthood, resulting in poorer interpersonal relationships, including reduced quality and quantity of friendships, fewer intimate relationships, and greater social withdrawal compared with full-term individuals (17). Furthermore, prolonged neonatal intensive care, exposure to stressful procedures, and early separation from caregivers may interfere with secure attachment, increasing vulnerability to later psychopathology. Additionally, elevated parental stress and overprotective parenting styles in families of preterm children may further contribute to adverse mental health outcomes. On the other hand, it is well established that good social functioning likely acts as a protective factor against psychopathology (20, 21). Therefore, social difficulties following preterm birth may transdiagnostically increase the risk of developing psychiatric disorders later in life.

The increased risk for deviations in neurodevelopmental trajectories in preterm individuals is thought to arise from a combination of biological and environmental factors related to premature exposure, during a critical window for synaptogenesis, dendritic arborization, cortical layering, and glial proliferation of the developing brain (22). In this context, studies of VPT individuals have reported reduced volumes in the orbitofrontal cortex, fusiform gyrus, amygdala, and hippocampus, alongside diminished integrity of the white matter tracts connecting these regions (8, 18). Such alterations may represent a neurodevelopmental substrate also for the later emergence of typically adult psychopathology. This framework is consistent for instance with the two-hit model of schizophrenia (23, 24), which posits that an early perinatal insult confers neuronal vulnerability, while a subsequent insult during critical developmental periods—particularly adolescence—leads to aberrant synaptic pruning and disrupted connectivity in key brain regions, ultimately contributing to disease onset.

In parallel, inflammatory processes implicated in the initiation of preterm birth may contribute to triggering inflammation-driven microglial activation, leading to altered synaptogenesis during critical developmental periods. This, in turn, may disrupt neuronal connectivity, representing a shared mechanism underlying ASD and other neurodevelopmental conditions (25–27). Furthermore, emerging evidence suggests that genetic and epigenetic factors may play a critical role in both the etiology of preterm birth and the long-term health outcomes of survivors (28).

The present study aimed to prospectively assess individuals born very preterm or extremely preterm for neurodevelopmental outcomes; second objective was to retrospectively investigate possible associations between early neurodevelopmental trajectories and specific psychopathological and functional outcomes in those individuals in adolescence. Particularly, since a key challenge in early identification is that overt psychiatric symptoms often do not emerge until childhood or later, this study aimed at investigating whether early motor and cognitive longitudinal predictors —collected during follow-up assessments in the first years of life—might be associated with a higher risk of later psychiatric conditions and functional impairment. Given the limited evidence on these long-term associations, the analyses should be considered exploratory, and no specific directional hypotheses were formulated.

## 2 Materials and methods

### 2.1 Design

This retrospective-prospective observational study was conducted at the University Hospital of Parma between December 2024 and April 2025, in compliance with the Declaration of Helsinki and local regulations. The study involved the retrospective retrieval of neonatal anamnestic information and early neurodevelopmental data, including Bayley-II assessment scores, from routine clinical records, together with the prospective collection of adolescent psychopathological and functional assessments. Ethical approval was obtained with Protocol Number 634.2024 which also authorized retrospective access to participants' clinical records for research purposes. Informed consent was obtained from all participants or their legal guardians, with the assent of minors. Inclusion also required the availability of early developmental data and consent for adolescent reassessment.

### 2.2 Participants

The study involved a cohort of 118 individuals born extremely preterm (EPT <28 weeks) and very preterm (VPT ≥28 weeks) between 2005 and 2011. In the first phase, after birth, neonatal clinical and anamnestic data were retrospectively collected, including sex, twin status, gestational age, birth weight, length of stay in the neonatal intensive care unit (NICU), and family history of neurological and psychiatric disorders. Early neurodevelopment was assessed within the first two years of life using the Bayley Scales of Infant and Toddler Development, Second Edition (Bayley-II). In the second phase, about fifteen years later, participants were contacted to undergo a multidimensional evaluation, which included self-administered questionnaires distributed via email through a secure web-based application.

### 2.3 Measures

All participants, together with one parent, completed a multidimensional psychopathological assessment, which included the Child Behavior Checklist (CBCL), the Autism Spectrum Quotient (AQ), the Obsessive–Compulsive Inventory (OCI), the Prodromal Questionnaire–16 (PQ-16) and the Inventory of Psychotic-Like Anomalous Self-Experiences (IPASE).

- Child Behavior Checklist (29, 30), part of the Achenbach System of Empirically Based Assessment (ASEBA) is a widely used questionnaire designed to assess emotional and behavioral functioning from both parental and self-report perspectives and internalizing and externalizing symptoms. CBCL comprises 113 corresponding items that cover a broad range of emotional and behavioral problems experienced in the past 6 months, which are displayed in eight syndrome scales. The items are rated from 0 (not true) to 2 (very true or often true). Adolescents fill out the Youth Self Report (YSR) while their parents complete the CBCL. Raw scores are converted into age- and sex-adjusted T-scores (M = 50, SD = 10) based on Italian and international norms.

The CBCL provides empirically derived syndrome scales, broadband scales (Internalizing, Externalizing, and Total Problems). T-scores below 65 indicate non-clinical functioning, scores between 65 and 69 indicate borderline functioning, and scores of 70 or above indicate clinically significant problems. The CBCL demonstrated high internal consistency, good test–retest reliability, convergent validity with other measures of child psychopathology (31, 32), and strong cross-cultural validity (33). Furthermore, a cross-informant agreement between CBCL and YSR scores was assessed using Intraclass Correlation Coefficients (ICCs) (34).

- Autism Spectrum Quotient (AQ) (35) is a 50-item self-report questionnaire designed to quantify autistic spectrum traits in individuals with cognitive functioning within the normal range. Participants aged <16 years completed the Autism Spectrum Quotient—Adolescent version (36), whereas participants aged ≥16 years completed the Autism Spectrum Quotient—Adult version. The AQ-Adolescent is completed by a parent or caregiver, whereas the AQ-Adult is a self-report questionnaire. The two versions are highly comparable, as both consist of 50 items assessing the same five domains of autistic traits: Social Skills, Attention to Detail, Attention Switching, Communication, and Imagination. Therefore, the two scales share underlying constructs, even though some items are slightly adapted to better reflect the adolescent's daily life context. Each item is scored dichotomously (0–1), yielding a total score ranging from 0 to 50, with higher scores indicating greater autistic traits. A cut-off score of > 30 has been shown to provide good discriminative accuracy for identifying individuals with clinically significant autistic traits and to ensure consistency in the assessment of adolescents and adults (37). To evaluate the impact of this choice, AQ prevalence was additionally calculated using the conventional clinical threshold of ≥32.The Italian version (38) demonstrated good psychometric properties and has been widely used in both clinical and non-clinical populations, including studies involving preterm-born individuals (36, 39, 40).
- Inventory of Psychotic-Like Anomalous Self-Experiences (IPASE) (41) is a 57-item self-report questionnaire assessing experiential self-disturbances conceptualized as a transdiagnostic dimension associated with psychosis risk. Items are grouped into five dimensions: Cognition, Presence, Consciousness, Somatization, and Transitivism. Each item is rated on a 5-point Likert scale (1–5), yielding a total score ranging from 57 to 285, with higher scores indicating greater severity of anomalous self-experiences. No specific clinical cut-off score has been established for the scale. The instrument shows high internal consistency and good convergent validity with measures of schizotypy and psychotic distress (41). The Italian version of the IPASE questionnaire showed good to excellent internal consistency and reliability (42).
- Obsessive Compulsive Inventory—Child Version (OCI-CV) (43, 44) consists of 21 items covering six symptom domains: Doubting/Checking, Obsessions, Washing, Ordering, Hoarding, and Neutralizing. Each item is rated on a 3-point Likert scale (0–2), yielding a total score ranging from 0 to 42, with higher scores indicating greater obsessive–compulsive symptom severity. A total score of 11 is considered indicative of clinically significant symptomatology (45). The OCI-CV has been validated in both clinical and community samples of children and adolescents aged 7 years and older, demonstrating good reliability and convergent validity.
- Prodromal Questionnaire—16 items (PQ-16) (46, 47) 16-item self-report screening questionnaire assessing anomalous perceptual experiences, ideas of reference/paranoia, cognitive disorganization, and negative symptoms. Items are answered dichotomously (true/false), and endorsed items are rated for associated distress on a 4-point Likert scale (0–3), yielding both a Symptoms Total Score (range: 0–16) and a Total Distress Score (range: 0–48). A Symptoms Total Score of ≥6 and a Total Distress Score of ≥8 are considered indicative of increased risk for psychosis and warrant further clinical assessment. The Italian version shows excellent psychometric properties in clinical and non-clinical samples and is widely recommended for psychosis risk screening in adolescents and young adults (47, 48).

Historical clinical data were reviewed from medical records, particularly focusing on early developmental scores obtained by the Bayley Scales of Infant and Toddler Development—Second Edition (Bayley-II) (49). The Bayley-II is designed to evaluate infants and young children aged 1 to 42 months and assesses developmental functioning across two domains: Cognitive Development, and Motor Development respectively expressed by Mental Development Index (MDI) and Psychomotor Developmental Index (PDI). The scale provides a comprehensive measure of early developmental outcomes and is widely used in both clinical and research settings. In our cohort, the Bayley Scales were administered longitudinally at three-month intervals from 3 to 24 months of age.

### 2.4 Statistical analysis

Due to the small sample size, both non-parametric and parametric methods were applied, as the normality of variable distributions could not be reliably assessed. After descriptive analysis of the sample, associations among continuous variables were examined using Spearman's rank correlations (*ρ*), with Benjamini-Hochberg false-discovery-rate correction applied within each family of correlations, while differences between categorical and continuous variables were tested using Welch's *t*-test or the Wilcoxon rank-sum test, selected per variable after a Shapiro–Wilk normality check. Analyses aimed at evaluating:

(i) Pattern of associations between psychopathological variables and neonatal clinical variables (i.e., gestational age, birth weight, NICU length of stay, sex, twin status, family history of neurological and psychiatric disorders).
(ii) Pattern of associations between psychopathological variables and early developmental quotient indices in the first 24 months of life.
(iii) Pattern of associations among psychopathological scores derived from self-report measures (AQ, IPASE, IPQ-16, OCI-CV, CBCL, and YSR).

Significant correlations were further explored using linear regression analyses to identify potential early predictors of later psychopathology. For all models, the trajectory intercept represented the estimated level of motor development (Bayley-II PDI) and cognitive development (Bayley-II MDI) at the reference age, while the slope represented the rate of change in motor and/or cognitive development over time during the first 24 months of life. The means of the latent intercept and slope terms were examined to describe the average level and average rate of change in motor and/or cognitive development across the entire sample. Variances of the intercept and slope terms were evaluated to assess the presence of inter-individual differences in baseline motor development and neurodevelopmental trajectories. Using the longitudinal growth model with the best-fitting functional form, neonatal clinical and anamnestic variables were tested as potential antecedents of neurodevelopment trajectories. These included demographic and medical characteristics (i.e., sex, gestational age, birth weight, twin status, length of NICU stay, and family history of neurological and psychiatric disorders), as well as indices of early developmental functioning derived from the Bayley-II. Each predictor was initially examined in separate unadjusted models, controlling for age at assessment. Predictors showing an association with either the intercept or slope term at a threshold of *p* < .15 [a liberal screening threshold recommended for candidate-predictor selection (50, 51)] were subsequently included in a single adjusted model. The adjusted model controlled *a priori* for gestational age at birth and sex.

To identify patterns of joint variation between early developmental outcomes and later psychopathology, we performed latent profile analysis (LPA) on standardized mean scores of PDI and MDI (averaged across time points), together with AQ, OCI, PQ-16, IPASE, and CBCL-AP.

Models with 1 to 4 profiles were estimated using a Gaussian mixture approach with varying variances and zero covariances. Model fit was evaluated using the Bayesian Information Criterion (BIC) as primary index, together with entropy, the Integrated Completed Likelihood (ICL), and the bootstrap likelihood ratio test (BLRT). The solution with the lowest BIC and adequate classification quality was retained. Given the modest sample size (*n* = 52) relative to the seven indicators, this solution should be regarded as exploratory: models with more profiles yielded marginally lower BIC values but produced small, unstable classes and were therefore not retained, the two-profile model being preferred for parsimony and interpretability.

## 3 Results

**Figure 1:**
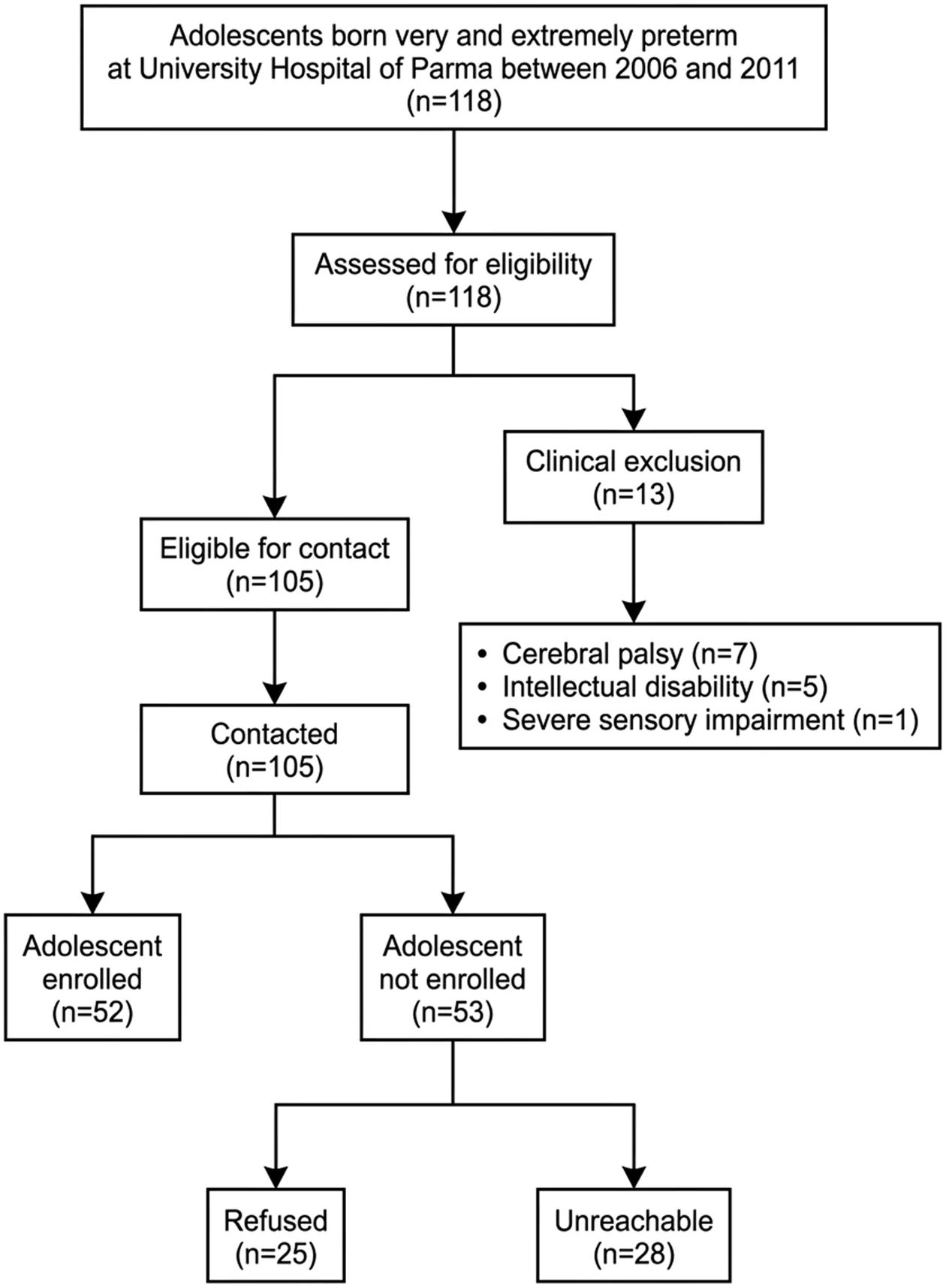
CONSORT-style flow diagram of participant selection.

The study involved a cohort of 52 adolescents aged between 14 and 18 years with a a mean age of 15.62 years (SD = 1.61) at the time of questionnaire administration, selected from an original group of 118 individuals born extremely preterm (EPT <28 weeks) and very preterm (VPT ≥28 weeks) between 2005 and 2011. A total of 66 patients were excluded from this stage because they presented with cerebral palsy, intellectual disability, severe sensory impairments, or because they were unreachable or declined participation in the study (Figure 1). The final sample underwent a multidimensional evaluation which included self-administered questionnaires. Sample characteristics are reported in Table 1. The mean gestational age was 28 weeks (SD = 1.8; range: 23–31 weeks); 32 participants were male (61.5%) and 20 were female (38.5%). The mean birth weight was 1,120.8 g (SD = 322.4).

**Table 1:** Anamnestic, neonatal and neurodevelopmental data stratified by gestational-age group.

Variable	Very preterm group (VPT) (*n* = 39)	Early preterm group (EPT) (*n* = 13)	Test	*p*	Effect size
Gestational age, wk	28.9 ± 0.9	25.5 ± 1.5	Wilcoxon	**<**.**001**	rb = 1.00
Birth weight, g	1,196.7 ± 307.2	809.8 ± 171.5	Welch	**<**.**001**	g = 1.37
NICU stay, d	54.1 ± 30.2	99.0 ± 42.5	Wilcoxon	.**003**	rb = 0.67
Male sex, *n* (%)	23 (59%)	9/13 (69%)	Fisher	.743	—
Twins, *n* (%)	17 (44%)	0/13 (0%)	Fisher	.005	—
Bayley-II MDI 3 months	94.9 ± 6.3	85.1 ± 17.7	Welch	.084	g = 0.94
Bayley-II MDI 6 months	94.6 ± 7.9	89.0 ± 9.2	Wilcoxon	.062	rb = 0.44
Bayley-II MDI 9 months	95.5 ± 8.6	91.1 ± 14.6	Welch	.368	g = 0.40
Bayley-II MDI 12 months	98.3 ± 9.7	86.5 ± 14.6	Welch	.**021**	g = 1.03
Bayley-II MDI 18 months	93.3 ± 10.4	81.4 ± 17.1	Welch	.124	g = 0.93
Bayley-II MDI 24 months	96.0 ± 13.1	82.6 ± 21.9	Welch	.258	g = 0.77
Bayley-II PDI 3 months	88.5 ± 7.4	83.9 ± 4.2	Wilcoxon	.100	rb = 0.32
Bayley-II PDI 6 months	85.3 ± 7.6	78.8 ± 19.1	Welch	.374	g = 0.57
Bayley-II PDI 9 months	90.5 ± 12.8	81.6 ± 18.6	Wilcoxon	.342	rb = 0.20
Bayley-II PDI 12 months	90.7 ± 12.9	81.8 ± 15.3	Welch	.096	g = 0.64
Bayley-II PDI 18 months	91.8 ± 9.7	84.5 ± 16.9	Welch	.282	g = 0.59
Bayley-II PDI 24 months	93.0 ± 8.8	80.0 ± 20.3	Welch	.187	g = 0.88

Continuous variables: mean ± SD; between-group test chosen per variable by Shapiro–Wilk (Welch t-test with Hedges' g, or Wilcoxon rank-sum with rank-biserial r). Bayley-II MDI, Bayley Scales of Infant and Toddler Development—Second Edition, Mental Development Index; Bayley-II PDI, Bayley Scales of Infant and Toddler Development—Second Edition, Psychomotor Developmental Index.

Bold values indicate statistically significant results (*p* < 0.05).

### 3.1 Comparisons between extremely preterm and very preterm groups

The sample was divided into two groups according to gestational age: extremely preterm (EPT <28 weeks) (*n* = 13; 25%) and very preterm (VPT ≥ 28 weeks) (*n* = 39; 75%). Considering gestational age, we observed higher cognitive scores, assessed through the MDI, in VPT infants compared with EPT infants with statistical significance for MDI scorers at 12 months (*p* = .018).

Moreover, VPT infants showed higher AQ scores (*p* = .044), whereas EPT infants presented higher CBCL-AP scores (*p* = .009). With respect to psychopathological measures, the total IPASE score showed a mean of 99.4 (SD = 39.0). On the OCI-CV, the mean total score in the sample was 7.9 (SD = 7.9). Furthermore 29 participants (55.7%) exceeded the study threshold of >30 on the AQ, whereas 26 (51.0%) exceeded the conventional clinical threshold of ≥32, indicating clinically autistic traits; additionally, 12 participants (23.1%) scored above 60 on the CBCL Attention Problems scale, consistent with ADHD-related features. Other CBCL and YSR scores were within the normative range, with higher values in internalizing domains (particularly withdrawn/depressed, somatic complaints, and anxiety) and lower scores in total competence and externalizing domains; all results are summarized in Table 2. Correlation between neonatal clinical–anamnestic variables and psychopathological measures.

**Table 2:** Psychopathological measures stratified by gestational-age group (mean ± SD), with between-group test and effect size.

Scale	VPT (*n* = 39)	EPT (*n* = 13)	Test	*p*	Effect size
CBCL Internalizing	54.7 ± 10.0	60.6 ± 12.0	Welch	.126	g = 0.56
CBCL Externalizing	47.4 ± 9.5	49.5 ± 9.5	Welch	.507	g = 0.21
CBCL Total	50.0 ± 10.4	55.8 ± 11.0	Welch	.107	g = 0.55
CBCL Social	53.9 ± 6.0	59.0 ± 10.7	Wilcoxon	.129	rb = 0.28
CBCL Thought	54.1 ± 5.6	57.5 ± 7.5	Wilcoxon	.194	rb = 0.24
CBCL Attention	54.6 ± 5.9	60.2 ± 7.8	Wilcoxon	.**005**	rb = 0.52
YSR Internalizing	52.9 ± 11.4	55.3 ± 11.3	Welch	.531	g = 0.21
YSR Externalizing	48.8 ± 9.0	46.5 ± 12.2	Welch	.551	g = 0.23
YSR Total	51.1 ± 10.1	52.8 ± 12.0	Welch	.662	g = 0.16
YSR Social	54.7 ± 6.4	58.8 ± 9.2	Wilcoxon	.168	rb = 0.26
AQ	32.3 ± 6.9	27.5 ± 6.6	Welch	.**044**	g = 0.69
OCI-CV	7.4 ± 7.5	9.3 ± 9.4	Wilcoxon	.815	rb = 0.05
PQ-16	4.1 ± 4.0	4.3 ± 3.7	Wilcoxon	.679	rb = 0.08
IPASE	98.3 ± 38.9	102.8 ± 41.2	Wilcoxon	.790	rb = 0.05

CBCL, child behavior checklist; YSR, youth self-report; AQ, autism spectrum quotient; OCI-CV, obsessive-compulsive inventory-child version; PQ-16, prodromal questionnaire-16; IPASE, inventory of psychotic-like anomalous self-experiences.

Bold values indicate statistically significant results (*p* < 0.05).

Gestational age was positively correlated with the total AQ score (*ρ* = .30, *p* = .033) and the YSR Social Problems subscale (*ρ* = −.280, *p* = .047). Gestational age was also positively correlated with mean motor development during the first two years of life (*ρ* = .30, *p* = .03), but not with the slope or intercept of the motor developmental trajectory (*ρ* = .07, *p* = .64; *ρ* = .25, *p* = .091, respectively). Birth weight was negatively correlated with the CBCL Anxiety Problems (*ρ* = −.40, *p* = .014), Attention Problems (*ρ* = −.38, *p* = .020), and ADHD Problems (*ρ* = −.35, *p* = .033) domains. Length of stay in the neonatal intensive care unit (NICU) was negatively correlated with the AQ total score (*ρ* = −.43, *p* = .020) and positively correlated with the YSR Social Problems domain (*ρ* = .39, *p* = .037). Length of stay was negatively correlated with birth weight (*ρ* = −.48, *p* < .001) and with the mean Bayley II motor development score across the first two years of life (*ρ* = −.45, *p* < .001), whereas no significant correlations were found with either the slope or the intercept of the motor developmental trajectory. The mean Bayley-II PDI score was negatively correlated with the Social Problems domain of both the CBCL (*ρ* = −.42, *p* = .002) and the YSR (*ρ* = −.36, *p* = .009). The intercept of the motor development trajectory was also negatively correlated with the Social Problems domain of the CBCL (*ρ* = −.43, *p* = .003) and the YSR (*ρ* = −.360, *p* = .009). Similarly, the slope of the motor development trajectory showed negative correlations with the Social Problems subscale of both the CBCL (*ρ* = −.360, *p* = .008) and the YSR (*ρ* = −.330, *p* = .031). In contrast, correlations with IPASE, OCI-CV, PQ-16, and with other CBCL/YSR domains were non-significant for all indices of motor development and trajectory. After Benjamini–Hochberg correction for multiple comparisons within each family, the associations of gestational age, birth weight and NICU length of stay with psychopathology did not remain significant and should be therefore interpreted as exploratory; the associations of early motor development (slope PDI and MDI) with adolescent Social Problems, and those among the psychopathological measures, survived correction.

### 3.2 Correlations among psychopathological variables

**Figure 2:**
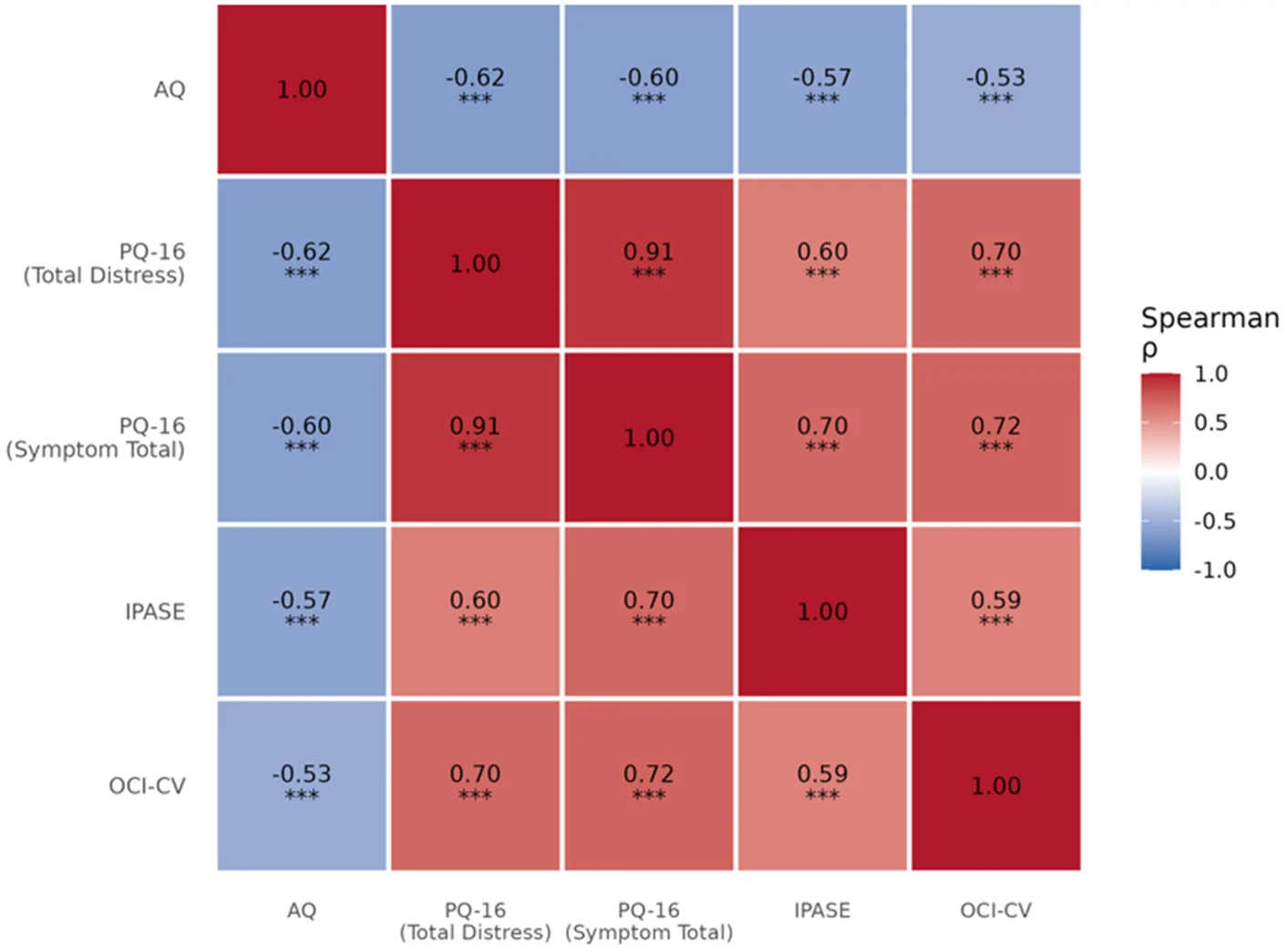
Heatmap of Spearman correlations among AQ, PQ-16 (Total Distress and Symptom Total scores), IPASE and OCI-CV. *N* = 52. AQ, autism spectrum quotient; PQ-16, prodromal questionnaire-16; IPASE, inventory of psychotic-like anomalous self-experiences; OCI-CV, obsessive-compulsive inventory–child version. All correlations survived Benjamini–Hochberg FDR correction (*** *q* < .001).

The AQ total score showed strong negative correlations with IPQ-16 scores (Total Distress: *ρ* = –.62; both *p* < .001), as well as with the all IPASE dimensions and OCI-CV (*ρ* = –.57 to –.65; all *p* < .001; Total Symptoms: *ρ* = –.62) (Figure 2).

Positive associations were also observed with CBCL scales (Externalizing Problems: *ρ* = .44, *p* = .001; Total Problems: *ρ* = .31, *p* = .027) and YSR scales (Internalizing Problems: *ρ* = .28, *p* = .044; Externalizing Problems: *ρ* = .42, *p* = .002; Total Problems: *ρ* = .45, *p* < .001). The total OCI-CV score was directly correlated with the YSR Internalizing Problems (*ρ* = .41, *p* = .003), Total Problems (*ρ* = .38, *p* = .005), and Thought Problems (*ρ* = .46, *p* < .001) domains. Weaker positive correlations were also observed with YSR Externalizing Problems (*ρ* = .27, *p* = .049) and Anxiety Problems (*ρ* = .31, *p* = .028). No significant correlations emerged between the total OCI-CV score and CBCL scales.

IPASE scores were also positively correlated with CBCL scales, specifically Externalizing Problems (*ρ* = .46, *p* < .001) and Total Problems (*ρ* = .40, *p* = .003), as well as with YSR scales, including Externalizing Problems (*ρ* = .40, *p* = .004), Total Problems (*ρ* = .37, *p* = .007), and Thought Problems (*ρ* = .48, *p* < .001). A weaker association was observed with the YSR Attention Problems domain (*ρ* = .35, *p* = .011). Similarly, the IPQ-16 Total Distress score demonstrated positive correlations with YSR Internalizing Problems (*ρ* = .52, *p* < .001), Total Problems (*ρ* = .45, *p* < .001), and Thought Problems (*ρ* = .54, *p* < .001). Additional weaker associations were found with YSR Externalizing Problems (*ρ* = .28, *p* = .040), Anxiety Problems (*ρ* = .34, *p* = .013), and Attention Problems (*ρ* = .33, *p* = .017), as well as with CBCL Externalizing Problems (*ρ* = .31, *p* = .027) and Total Problems (*ρ* = .29, *p* = .036). Consistent with the pattern of results described above, AQ scores were negatively correlated with CBCL Total Problems (*ρ* = −.32, *p* = .022) and Thought Problems (*ρ* = −.27, *p* = .047), as well as with YSR Internalizing Problems (*ρ* = −.35, *p* = .011), Total Problems (*ρ* = −.33, *p* = .016), Social Problems (*ρ* = −.33, *p* = .019), Thought Problems (*ρ* = −.45, *p* < .001), and Attention Problems (*ρ* = −.28, *p* = .042). Furthermore, Intraclass Correlation Coefficients (ICCs) indicated an overall poor-to-fair level of agreement between parent (CBCL) and adolescent (YSR) reports. Fair agreement was observed for the Total Problems (ICC=0.582, 95%CI=[0.297,0.762], p<0.001), Internalizing scales (ICC=0.510, 95%CI=[0.183,0.719], p<0.001), Social Problems (ICC=0.456, p=0.004), and Attention Problems (ICC=0.490, p=0.002); whereas agreement for the Externalizing scale was poor (ICC=0.316, 95%CI=[−0.015,0.589], p=0.030). At the syndrome level, agreement ranged from poor to fair across most domains, while no significant agreement was found for the Withdrawn/Depressed and Rule-Breaking Behavior scales.

### 3.3 Early neurodevelopmental scores and longitudinal trajectories

**Figure 3:**
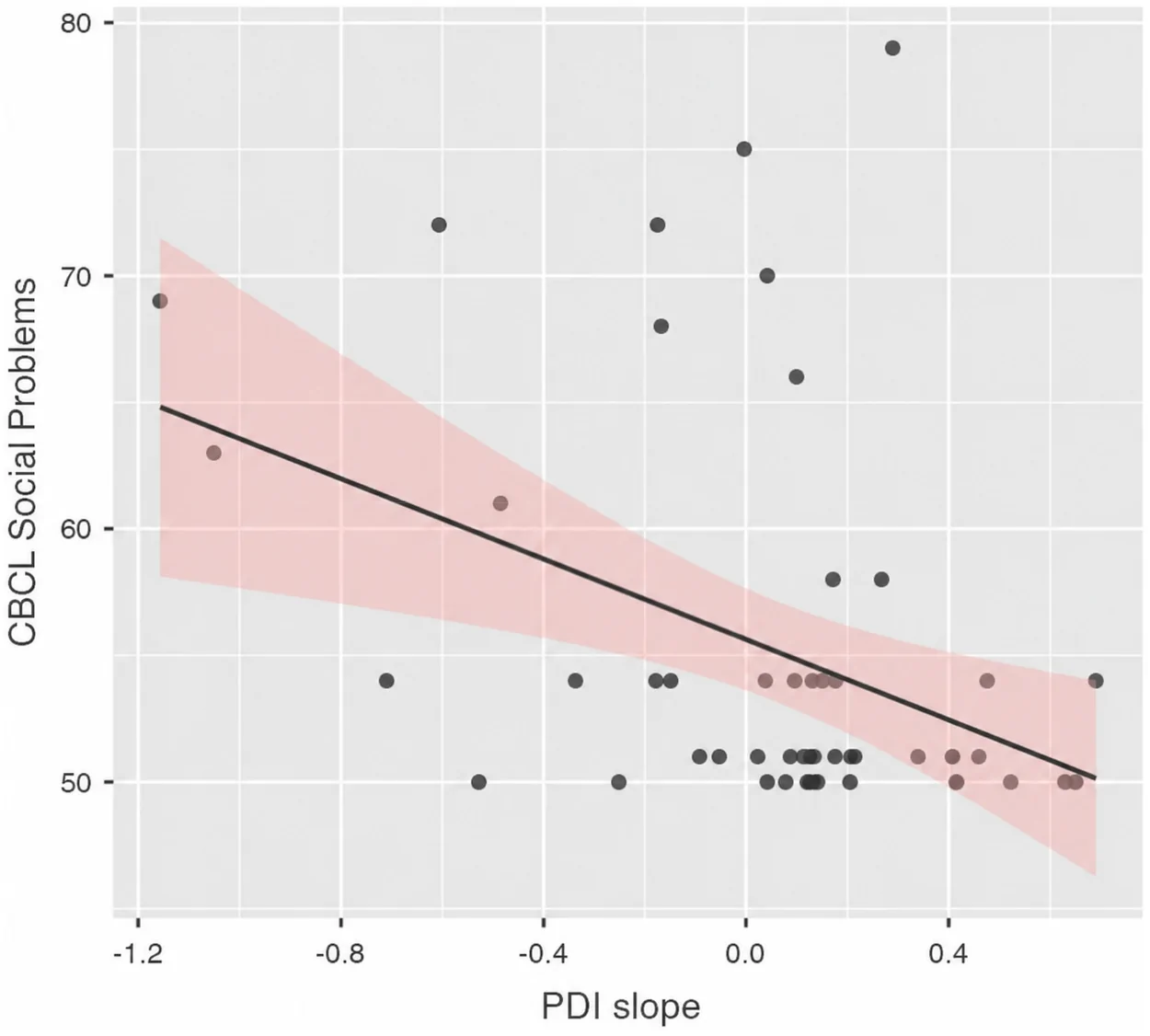
Scatter plot showing the correlation between PDI slope and CBCL Social Problems scores in the study population.

The mean Bayley PDI score was strongly correlated with the intercept (*ρ* = .85, *p* < .001), indicating that children with lower initial motor scores tended to maintain lower average levels of motor performance over time. A significant positive correlation also emerged between the mean Bayley PDI score and the slope (*ρ* = .67, *p* < .001), suggesting that steeper motor growth trajectories were associated with higher average levels of motor development (Figure 3). Finally, the intercept and slope showed a positive correlation of smaller magnitude (*ρ* ≈ .31, *p* = .024), indicating that higher initial motor levels were associated, albeit to a lesser extent, with faster motor growth over time. Significant associations emerged between MDI parameters and indices of social functioning. Specifically, the MDI intercept was negatively correlated with social problems as measured by the Child Behavior Checklist (CBCL; *ρ* = −0.331, *p* = .017) and the Youth Self-Report (YSR; *ρ* = −0.424, *p* = .002). Similarly, the MDI slope showed significant negative correlations with CBCL Social Problems (*ρ* = −0.346, *p* = .012) and YSR Social Problems (*ρ* = −0.413, *p* = .003). In contrast, the MDI slope was positively correlated with total obsessive–compulsive symptoms as assessed by the Obsessive-Compulsive Inventory—Child Version (OCI-CV Total; *ρ* = 0.279, *p* = .048). Finally, the mean MDI score across the 3–24-month interval was negatively correlated with both CBCL Social Problems (*ρ* = −0.301, *p* = .030) and YSR Social Problems (*ρ* = −0.415, *p* = .002).

### 3.4 Latent profile analysis

**Figure 4:**
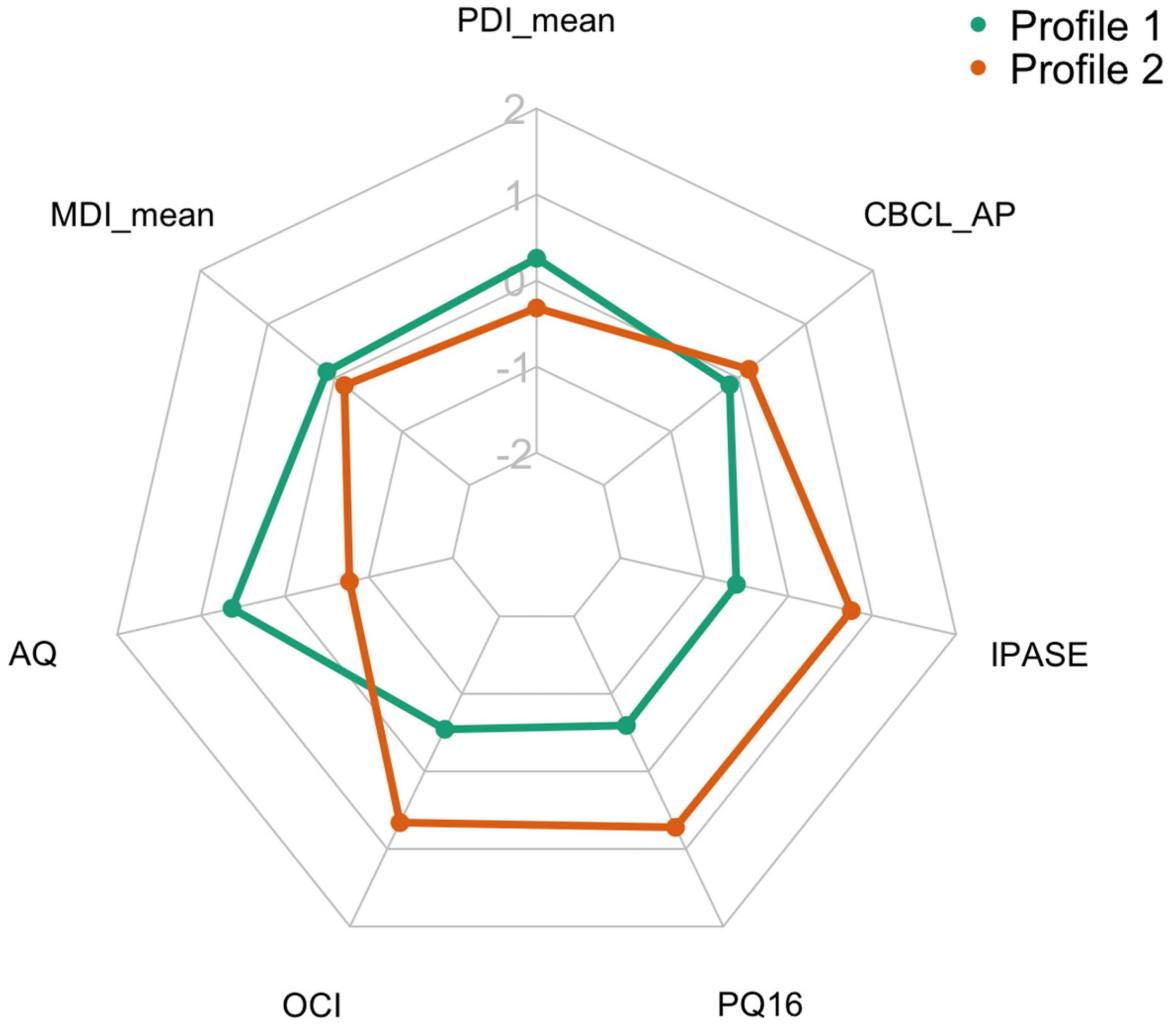
Radar plot of the latent profiles of early developmental and psychopathological dimensions.

LPA supported a two-profile solution (Figure 4) as the best-fitting model according to the Bayesian Information Criterion (BIC = 949). Classification quality was high, with entropy = 0.924, and excellent posterior assignment probabilities (minimum = 0.979; maximum = 0.980). Moreover, profile sizes were reasonably balanced (Profile 1 = 45% of the sample; Profile 2 = 55%). Profile 1 (“High autism traits”) was characterized by high AQ scores and relatively low scores on OCI, PQ-16, and IPASE. On the other hand, Profile 2 (“High psychotic and OCD traits”) showed, with markedly elevated OCI, PQ-16, and IPASE scores. The two profiles did not differ significantly in PDI [*χ*^2^(1) = 2.65, *p* = .103] and MDI [*χ*^2^(1) = 0.116, *p* = .733] means. Similarly, the two profiles did not differ in the CBCL-AP [*χ*^2^(1) = 2.36, *p* = 0.124]. Participants classified in Profile 2 exhibited significantly lower autistic traits compared to Profile 1, as shown by a highly significant effect on the AQ total score [F(1,49) = 48.16, *p* < .001]. Profile 2 showed significantly higher OCD symptoms [OCI: *χ*^2^(1) = 16.04, *p* < .001], and psychotic traits [PQ-16: χ^2^(1) = 22.25, *p* < .001; IPASE: *χ*^2^(1) = 23.42, *p* < .001] (Table 3). Finally, to directly test whether the two latent profiles could differ in CBCL Social Problems, we used the Mann–Whitney U test, given non-normality in both profiles (Shapiro–Wilk *p* < .001 in both). Profile 1 (*n* = 28; M = 53.21, SD = 5.56) and Profile 2 (*n* = 23; M = 57.74, SD = 9.25) did not differ significantly in CBCL Social Problems (W = 252, *p* = .177, rank-biserial r = .22), indicating that the latent profiles are not further distinguished by parent-reported social difficulties.

**Table 3:** Descriptive statistics for the latent profiles.

Profile	PDI (mean ± SD)	MDI (mean ± SD)	AQ (mean ± SD)	OCI (mean ± SD)	PQ16 (mean ± SD)	IPASE (mean ± SD)	CBCL_AP (mean ± SD)
1	89.74 ± 4.92	94.99 ± 4.98	35.64 ± 4.58	3.57 ± 2.76	1.82 ± 1.36	75.25 ± 18.43	54.96 ± 6.34
2	84.30 ± 12.43	92.58 ± 12.71	25.69 ± 5.65	13.13 ± 9.03	6.95 ± 4.16	128.73 ± 37.46	56.91 ± 7.21

### 3.5 Predictive role of neonatal factors and motor-cognitive development on social functioning

Hierarchical multiple regressions were conducted to investigate the predictive role of neonatal factors and early motor and cognitive development on later social functioning.

A first regression analysis was performed to examine whether PDI slope explained additional variance in CBCL Social Problems (dependent variable) beyond gestational age. At Step 1, gestational age significantly predicted CBCL Social Problems [R = .378, R^2^ = .143 (adjusted R^2^ = .126), F(1, 50) = 8.354, *p* = .006]. Gestational age was negatively associated with social problems (B = −1.578, *β* = −.378, t = −2.890, *p* = .006), indicating that higher gestational age was associated with fewer social problems. At Step 2, the PDI slope was entered into the model. The overall model remained significant [R = .464, R^2^ = .215 (adjusted R^2^ = .183), F(2, 49) = 6.705, *p* = .003]. In the model, the PDI slope resulted in an increase in explained variance (ΔR^2^ = .072), accounting for an additional 7.2% of variance in CBCL Social Problems. PDI slope emerged as the only significant negative predictor (B = −5.782, *β* = −.289, t = −2.115, *p* = .040), indicating that a more favorable motor developmental trajectory predicted fewer social problems in adolescence. A percentile bootstrap (5,000 resamples) gave a 95% CI for the standardized -PDI coefficient (β) of [−0.67, 0.02], which includes zero; this predictor should therefore be interpreted with caution despite reaching conventional significance.

A second regression analysis was conducted to examine whether MDI slope explained additional variance in CBCL Social Problems (dependent variable) beyond gestational age. Step 1 was identical to the first model reported above [gestational age: R^2^ = .143, F(1, 50) = 8.354, *p* = .006]. At step 2, the MDI slope was entered into the model. The overall model was significant [R = .427, R^2^ = .182 (adjusted R^2^ = .149), F(2, 49) = 5.452, *p* = .007], however neither predictors were statistically significant (Gestational age: B = −1.023, *β* = −.245, *p* = .122; MDI slope: B = −7.785, *β* = −.238, *p* = .133).

## 4 Discussion

The present study aimed to identify neurodevelopmental outcomes and early predictors of later psychopathology in a cohort of adolescents who were born VPT or EPT. The results, although preliminary, provide evidence that very and extremely preterm birth is associated with a substantial burden not only during the perinatal period, but also in the long term, including increased neurodevelopmental disorders and an elevated risk for psychopathological vulnerabilities in adulthood. Particularly, while in childhood the profile of impairments of individuals born preterm typically reflects the preterm behavioral phenotype, in adolescence and young adulthood prematurity was associated with an increased risk of a wide range of psychiatric disorders.

### 4.1 Preterm birth and neurodevelopment

In our sample, VPT infants showed higher cognitive scores on the MDI than EPT infants, with statistically significant differences at 12 months. These differences in gestational age may reflect distinct neurodevelopmental trajectories: VPT infants (*n* = 39; 75%) displayed higher AQ questionnaire scores, suggesting increased vulnerability to autistic traits, whereas EPT infants (*n* = 12; 25%) showed higher CBCL Attention Problems scale scores, indicating greater attention deficits, consistent with ADHD-related features.

As to ADHD profile, existing literature consistently reports a two- to three-fold increased risk for ADHD in VPT children (52), and up to a four-fold increased risk for ADHD in extremely preterm individuals (53, 54). Overall, in our sample 23.1% of patients scored above 60 on the CBCL Attention Problems scale. These findings also align with recent studies reporting that children born very preterm have up to a five-fold increased risk of attention-deficit/hyperactivity disorder compared with term-born peers (55). The higher prevalence of ADHD—predominantly of the inattentive subtype—represents only one of the manifestations of the preterm behavioural phenotype, which is further characterized by an increased risk of emotional disorders and autism spectrum disorder (12, 56).

Increased risk of autism spectrum disorder (ASD) associated with lower gestational age is also well established in literature, although only a few studies have directly compared EPT and VPT groups (13, 57). In our sample, 29 participants (55.7%) exceeded the cut-off score of 30 on the AQ questionnaire, indicating clinically significant autistic traits. Therefore, we identified autistic vulnerability in more than half of our patients, a much higher prevalence with respect to those reported in other studies (58). Previous studies (8, 13, 59, 60) for example have reported a risk for the autism spectrum up to 12 times higher among individuals born preterm compared with term-born controls. This discrepancy may be due to the fact that we did not focus on all preterm children, but specifically on those born extremely or very preterm, in whom the incidence is known to be higher. Moreover, autistic risk was assessed through a widely used, self-administered questionnaire (AQ), which, however, may have over-estimated behaviors (such as social withdrawal), which are inherently multi-factorial in the adolescent population; this is even more true since we decided to use the borderline AQ cutoff of 30. Further studies are therefore needed to investigate autistic traits in this population, which actually appear to be qualitatively different from those observed in idiopathic autism, as they appear to be mediated by underlying specific cognitive impairments, including distractibility and inattention (8).

### 4.2 Preterm birth and psychopathology

As to psychosis risk, 6 participants (11.5%) met the CBCL Thought Problems cut-off of ≥ 67, suggestive of psychotic vulnerability. By contrast, no significant scores were observed on the 16- item Prodromal Questionnaire (PQ-16) and on the Inventory of Psychotic-Like Anomalous Self-Experiences (IPASE). This discrepancy may be due to the limited sensitivity of these instruments within this age range, with respect to the Thought Problems Scale of CBCL, which, conversely has been found to be a diagnostically reliable screening tool for psychotic disorders in help-seeking and non-clinical youth populations (61). Direct comparison with the existing literature is challenging, as prior studies have predominantly focused on mood and anxiety disorders, while psychotic outcomes—aside from schizophrenia—are rarely examined (11).

With respect to obsessive–compulsive vulnerability, 14 participants (26.9%) reached the OCI cut-off score of 11 in line with previous evidence documenting a 2.6-fold increase in obsessive- compulsive disorder (OCD) risk (62). This finding corroborates the evidence that early-onset obsessive-compulsive symptoms may “cover” heterogeneous underlying neurodevelopmental perturbations (63), with psychopathology arising both in childhood (e.g., autism spectrum, ADHD) and in adulthood (e.g., affective and non-affective psychoses) (64–66).

### 4.3 Developmental trajectories

Previous studies (67) suggest that early-life inattention and autism spectrum disorder–related symptoms may represent prodromal manifestations of psychosis emerging along a continuum of psychopathological trajectories during the transition to adulthood. However, we failed to find direct significant associations between autistic traits (AQ) and psychotic vulnerability (IPASE, PQ-16) in our sample. Instead, and remarkably, we found a strong inverse relationship between autistic indexes (AQ) and psychotic indexes (both PQ-16 and IPASE), rather suggesting a “diametrical” model of autism and schizophrenia, which would posit the two phenotypes at the opposite ends of shared evolved adaptive traits (68).

In contrast, Obsessive–compulsive symptoms (OCI-CV) were directly associated with psychotic risk, as measured by both the PQ-16 and IPASE, further supporting the hypothesis that obsessive compulsive symptoms may represent early biomarkers of vulnerability to psychosis (63). Instead, these symptoms showed no association with autistic traits. This finding is consistent with previous literature indicating that individuals born preterm often present a phenotype characterized more by social communication difficulties than by repetitive, stereotyped or obsessive compulsive-like behaviors (21, 52).

Latent profile analysis (LPA), though preliminary due to the small sample size, would confirm the present findings, by identifying two distinct (“autistic” and “psychotic”) neurodevelopmental profiles. Particularly, the two distinct phenotypes found were not differentiated by overall motor and cognitive development, (Psychomotor Development Index (PDI) and Mental Development Index (MDI), nor in general behavioral problems and social problems as measured respectively by the CBCL-AP and the CBCL—social problems. Rather, the profiles were primarily distinguished by psychopathological features. Profile 1 (“autistic”) was characterized by higher autistic traits and low levels of obsessive–compulsive and psychotic symptoms in the context of relatively preserved developmental functioning. In contrast, Profile 2 (“psychotic”) was marked by significantly elevated psychotic features and obsessive-compulsive symptoms, alongside normal developmental scores and lower levels of autistic traits. Crucially, the finding that the “psychotic” profile exhibited lower autistic traits would further suggest that autistic traits *per se* do not constitute a risk factor for psychosis in this population. Instead, vulnerability to psychosis appears to cluster with obsessive–compulsive symptomatology, rather than with autism spectrum–related traits. Taken together, we speculate that distinct neurodevelopmental pathways may underlie autistic and psychotic phenotypes.

### 4.4 early predictors of social functioning

We aimed at investigating early predictors of later social dysfunction in VPT and EPT individuals, taking advantage of the long-term follow-up available in our cohort. Specifically, we focused on perinatal parameters and early psychomotor development, as assessed by the Bayley Scales in the first years of life, in line with previous literature (69, 70), while the domain 'social problems' of the CBCL was used as index of social outcome in adolescence.

Our findings would highlight the relevance of early motor development as a core component of later social impairment in children born preterm. Poorer PDI was the only predictor of CBCL social problems in adolescence, suggesting that early motor difficulties may be associated with later socio-behavioral functioning.

This association is consistent with the notion that motor development is closely intertwined with broader developmental domains, including social interaction and emotional regulation (71). Moreover, in our sample a strong association was found between the mean Bayley PDI score and the intercept of motor developmental trajectories, such that children with lower initial motor functioning tended to maintain lower average levels of motor performance over time. In addition, the positive association between the mean PDI score and the slope of motor developmental trajectory would suggest that steeper motor growth trajectories were linked to higher overall levels of motor development. Together, these findings hint at a relative stability of individual differences in motor functioning across early development, with early motor delays potentially marking a persistent vulnerability of social impairment.

Notably, the association with motor functioning observed in our study appears to extend previous findings, which have primarily linked greater cognitive risk in childhood to poorer social functioning in early adulthood. Although there is evidence that individuals born preterm are at increased risk for motor impairments, ranging from cerebral palsy to more subtle coordination difficulties (72, 73), the potential role of early motor functioning as a predictor of later socio-behavioural outcomes has received comparatively less attention. The present findings suggest that preterm birth may alter neural circuitry underlying early motor markers, shared by both autism and psychosis developmental trajectories (21, 74). Within this framework, the acquisition of motor skills in infancy scaffolds complex interactions with the physical and social environment, with potential associations across multiple domains of development (75).

Early motor difficulties may in fact impair self-embodiment processing, leading to an unstable and attenuated sense of self with blurred self-other boundaries, which is a core feature of schizophrenia vulnerability (76, 77). In a similar vein, motor impairments are highly prevalent in autism spectrum disorder, where they emerge early in development, often preceding socio-communicative difficulties (78–80). Coherently, deficits in sensorimotor representation of self- and other-generated actions have been frequently reported in preterm children (81, 82). Altogether, our results would support the evidence that early motor functioning may represent a sensitive marker for identifying individuals at increased vulnerability to autism (78) and psychosis (83).

## 5 Strengths and Limitations

A major strength of our study lies in the early and long-term follow-up of the cohort. Unlike previous studies relying on hospital discharge records for psychiatric diagnosis, our cohort was assessed in early infancy and monitored over time within clinical developmental services. Participants were never formally discharged and were re-contacted for the follow-up. This unique aspect allowed us to correlate early developmental data—particularly related to motor and cognitive milestones—with later psychiatric outcomes, offering valuable insight into potential early predictors of psychopathology.

The present findings should be interpreted in light of several limitations. First, the overall sample size, particularly the relatively small EPT subgroup, limited the statistical power of between-group comparisons, increasing the risk of both Type I and Type II errors. The modest sample size may also have affected the stability of the latent profile solution and the robustness of the regression analyses; therefore, the Latent Profile Analysis should be considered exploratory, and the identified profile solution should be regarded as hypothesis-generating, pending replication in larger, independent samples. Second, the use of self-report questionnaires provides information on psychopathological vulnerabilities and putative developmental trajectories rather than formal psychiatric diagnoses. Third, developmental functioning was assessed using the Bayley Scales of Infant Development, Second Edition (Bayley-II), which yields a single Developmental Quotient and does not distinguish between gross and fine motor development. Finally, the exclusion of individuals with cerebral palsy, intellectual disability, and severe sensory impairments, although necessary to ensure the feasibility and validity of the neuropsychological assessment, may have introduced selection bias by yielding a healthier-than-representative sample. Consequently, the prevalence of neurodevelopmental difficulties may have been underestimated, and the observed associations may have been attenuated.

## 6 Conclusions

In summary, individuals born very and/or extremely preterm, despite sharing similar perinatal and early developmental profiles, seem to follow distinct neurodevelopmental trajectories, leading to autistic or psychotic phenotypes. Moreover, early motor development emerged as a significant predictor of later social functioning difficulties in adolescence, regardless of the underlying developmental trajectory. Further research is needed to clarify the role of motor skills in the early identification of altered neurodevelopmental pathways, in order to provide a fine-grained motor assessment for personalized therapeutic intervention strategies. Moreover, longitudinal studies should be addressed to clarify the role of motor skills in the early identification of altered neurodevelopmental pathways. The present findings provide a potential rationale for early motor interventions in improving later developmental outcomes.

## Data Availability

The raw data supporting the conclusions of this article will be made available by the authors, without undue reservation.
